# Predictors of Appropriate Implantable Cardioverter-Defibrillator Intervention in Adults with Congenital Heart Disease

**DOI:** 10.3390/jcm15187128

**Published:** 2026-09-14

**Authors:** Jakub Malinowski, Łukasz Nowotka, Jakub Kosma Rokicki, Olgierd Woźniak, Paweł Syska, Michał Lewandowski, Artur Oręziak, Joanna Zakrzewska-Koperska, Patryk Marchewka, Anna Jargieło, Dariusz Zając, Błażej Kozłowski, Piotr Hoffman, Mariusz Pytkowski, Łukasz Szumowski, Ewa Warchoł-Celińska, Maciej Sterliński, Mirosław Kowalski

**Affiliations:** 1 Students’ Scientific Society, National Institute of Cardiology, 04-628 Warsaw, Poland; 2 Faculty of Medicine, Medical University of Warsaw, 02-091 Warsaw, Poland; 3 Department of Medical Informatics and Telemedicine, Medical University of Warsaw, 00-581 Warsaw, Poland; jakub.rokicki@wum.edu.pl; 4 First Department of Cardiology, Medical University of Warsaw, 02-097 Warsaw, Poland; 5 Department of Congenital Heart Diseases, National Institute of Cardiology, 04-628 Warsaw, Polandewarchol@ikard.pl (E.W.-C.);; 6 2nd Department of Arrhythmia, Arrhythmia Centre, National Institute of Cardiology, 04-628 Warsaw, Poland; psyska@ikard.pl (P.S.);; 7 1st Department of Arrhythmia, Arrhythmia Centre, National Institute of Cardiology, 04-628 Warsaw, Poland

**Keywords:** congenital heart disease, ventricular arrhythmias, implantable cardioverter-defibrillator, atrial fibrillation, sudden cardiac death, risk stratification

## Abstract

**Background:** Evidence to guide risk stratification after ICD implantation in adults with congenital heart disease (ACHD) is limited. We sought predictors of appropriate ICD intervention in this population. **Methods:** Consecutive ACHD patients implanted with an ICD (2009–2023) were analysed retrospectively. Time to first appropriate ICD intervention was modelled from a single, consistently defined start of observation. Because only 24 events were available, candidate predictors were pre-specified and each model limited to two covariates; sparse predictors used Firth penalisation, and multiplicity was addressed by the Benjamini–Hochberg false discovery rate procedure. **Results:** Among 56 patients (73.2% male; 22 primary and 34 secondary prevention), appropriate intervention occurred in 24 (42.9%) over a median follow-up of 51.9 months. Atrial fibrillation (HR 2.75, 95% CI 1.22–6.23) and radiographic cardiomegaly (HR 3.99, 95% CI 1.28–12.40) showed the largest unadjusted associations, whereas individual congenital lesions and disease-severity descriptors did not. No predictor remained significant after Benjamini–Hochberg correction (q ≈ 0.10). The very high hazard ratios for sparsely represented thyroid disorders (hypothyroidism, *n* = 2; hyperthyroidism, *n* = 4) were unstable under penalised estimation and are reported as exploratory. A pre-specified two-variable model (atrial fibrillation plus radiographic cardiomegaly) showed modest discrimination (optimism-corrected Harrell’s C-index 0.63). **Conclusions:** In a heterogeneous real-world ACHD cohort, a history of atrial fibrillation and radiographic cardiomegaly were associated with appropriate ICD intervention. Given the small number of events and the sparsity of several predictors, these findings should be regarded as hypothesis-generating and require validation in larger multicentre ACHD cohorts.

## 1. Introduction

Complex ventricular arrhythmias (VAs) commonly occur in patients with structural heart disease and may lead to sudden cardiac death (SCD) [1]. Congenital heart disease is increasingly prevalent in the adult population and, particularly in severe or complex forms, is associated with worsening heart failure and ventricular arrhythmias [2].

Implantable cardioverter-defibrillator (ICD) implantation remains the gold standard for the prevention of SCD in patients who have survived, or are at high risk of, sudden cardiac arrest [3]. Indications for ICD implantation in congenital heart disease in adults (ACHD) are largely based on expert consensus; consequently, there is limited evidence to support reliable SCD risk stratification in this population [4].

This study aimed to identify predictors of appropriate ICD intervention in patients with ACHD following ICD implantation.

## 2. Methods

### 2.1. Study Population

Consecutive patients with a diagnosis of ACHD who underwent ICD implantation for primary or secondary prevention between January 2009 and December 2023 were included in this single-centre, retrospective study. Several ICD models from four manufacturers were implanted: Medtronic (Minneapolis, MN, USA), Biotronik (Berlin, Germany), Abbott (Abbott Park, IL, USA) and Boston Scientific (Marlborough, MA, USA), at the operator’s discretion. Analysed parameters comprised demographic, clinical, electrocardiographic, and echocardiographic data, as well as blood test and chest radiography results, obtained at the start of observation (at the time of device implantation or at the first available subsequent record). The follow-up period extended until the last available clinical record, death, or heart transplantation.

### 2.2. Data Collection

During follow-up, the following data were collected: periprocedural complications related to ICD implantation requiring prolonged hospitalisation or intervention; long-term device-related adverse events, such as infections or lead failure; both appropriate and inappropriate ICD interventions reported during device interrogation; and electrical parameters of the defibrillation lead obtained at the final device interrogation during follow-up. All device interrogations were reviewed by experienced electrophysiologists; an intervention (shock or antitachycardia pacing) was adjudicated as appropriate when delivered for ventricular tachycardia or ventricular fibrillation and inappropriate when delivered for any other reason, most commonly supraventricular arrhythmia or oversensing. Radiographic cardiomegaly was defined on the chest radiograph as a cardiothoracic ratio exceeding 50%.

The study population was divided into two subgroups: patients who experienced appropriate ICD intervention during follow-up and those who did not.

### 2.3. Statistical Analysis

Categorical variables are presented as counts and percentages and continuous variables as medians with interquartile ranges (IQR, Q1–Q3); baseline comparisons used the chi-squared or Fisher’s exact test and the Wilcoxon rank-sum test, with effect sizes and 95% confidence intervals reported and baseline *p*-values treated as descriptive rather than as a screen for predictors. The primary endpoint was time to first appropriate ICD intervention, measured from a single, consistently defined start of observation (the time of device implantation or the first available record for patients implanted earlier elsewhere); interventions recorded at or before the start of observation were assigned a small positive offset, and prevalent devices were additionally handled with left-truncated (delayed-entry) models. Because only 24 events were available, candidate predictors were pre-specified on clinical grounds and the number of covariates in any model was limited to a maximum of two (events per variable ≥ 8–10); variables were not selected on the basis of univariable significance. Univariable associations were estimated with Cox proportional hazards models, reporting the hazard ratio, 95% CI, *p*-value, number of non-missing observations, number exposed, and number of events among the exposed. Sparse binary predictors (fewer than five exposed patients) were analysed with Firth-penalised Cox regression and treated as exploratory. Two pre-specified parsimonious multivariable models were fitted, and a ridge-penalised model with bootstrap optimism correction was used to assess stability and overfitting. The proportional hazards assumption was examined with Schoenfeld residuals; where it was violated, follow-up was split to characterise time-varying effects. Discrimination was summarised with the optimism-corrected Harrell’s C-index and with time-dependent AUC at 1, 3 and 5 years, rather than with ordinary ROC curves, which are not valid for censored time-to-event data. Multiplicity was addressed with the Benjamini–Hochberg false discovery rate procedure across the pre-specified predictors. Death and heart transplantation were treated as competing events in sensitivity analyses (cause-specific Cox and Fine–Gray models), and further sensitivity analyses excluded sparsely represented and influential observations; the number of observations and events is reported for every model, and no patients were excluded without explanation. Analyses were performed in R version 4.5.0 (2025-04-11; survival, coxphf, timeROC, cmprsk/riskRegression and rms packages); a two-sided *p*-value < 0.05 was considered statistically significant and was interpreted in the light of the multiplicity correction.

## 3. Results

### 3.1. Baseline Characteristics

**Figure 1:**
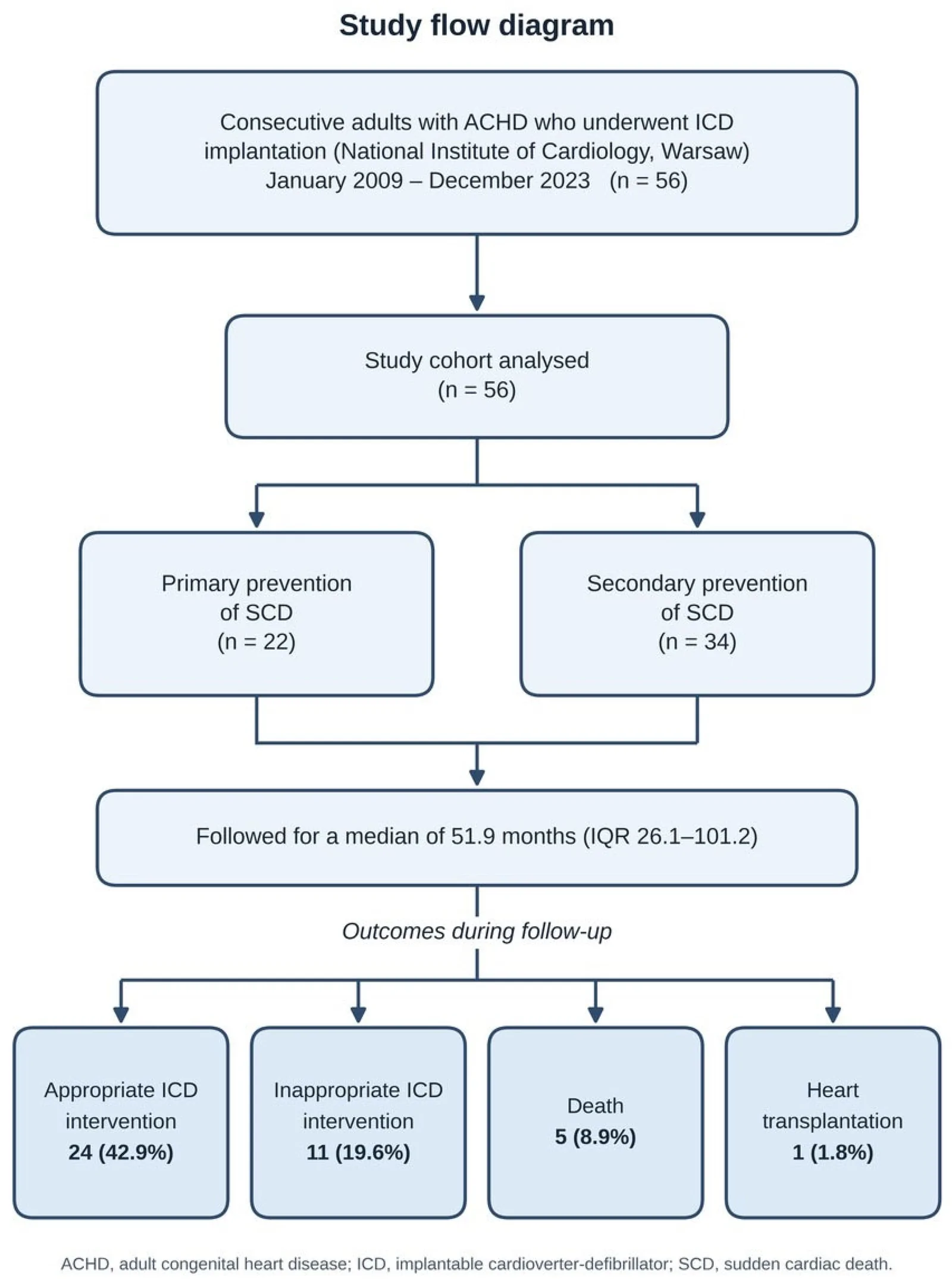
Study flow diagram.

**Figure 2:**
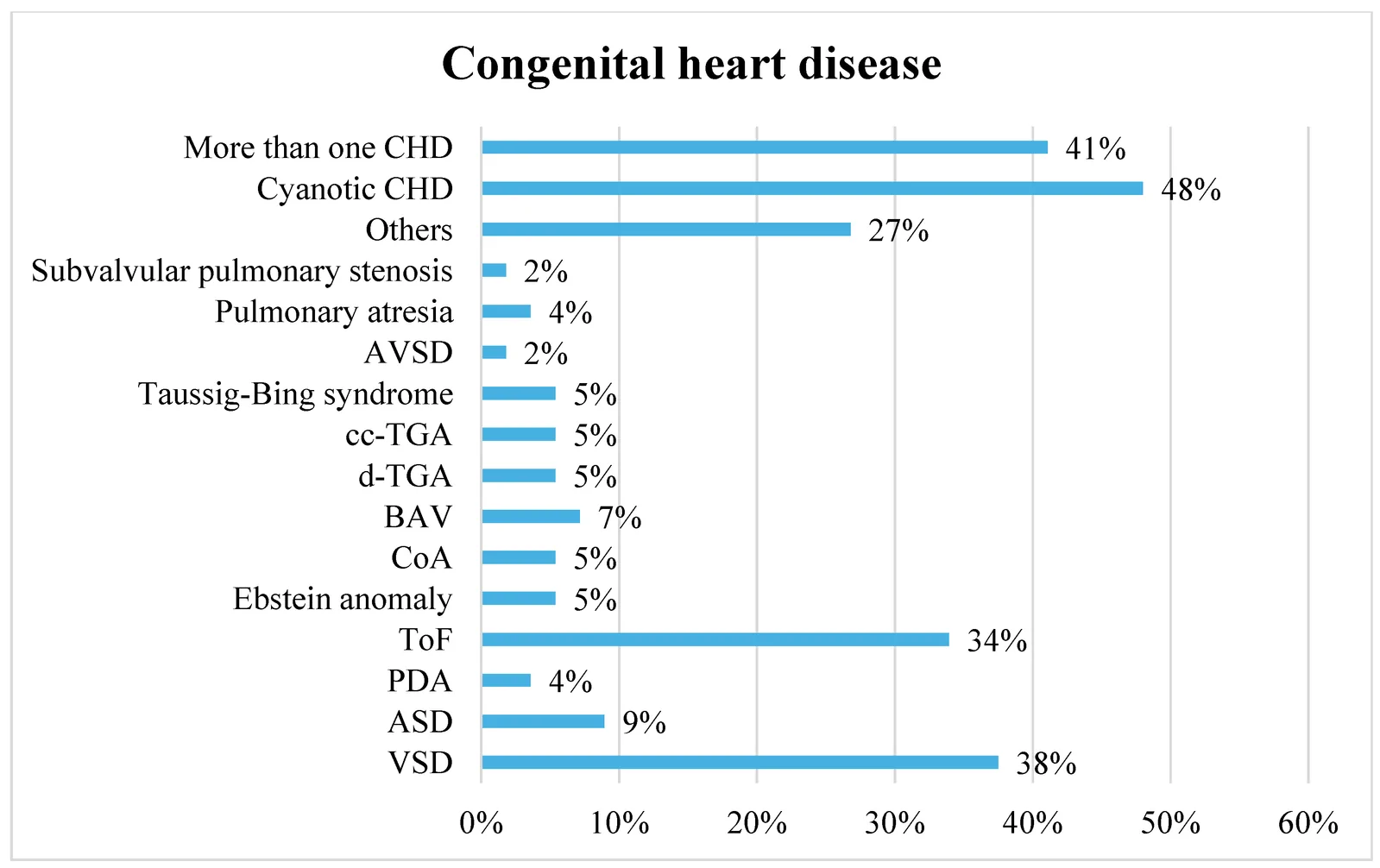
Congenital heart diseases in the study population. Abbreviations: ASD—Atrial Septal Defect; AVSD—Atrioventricular Septal Defect; BAV—Bicuspid Aortic Valve; cc-TGA—Congenitally Corrected Transposition of the Great Arteries; CHD—Congenital Heart Disease; CoA—Coarctation of the Aorta; d-TGA—Dextro-Transposition of the Great Arteries; PDA—Patent Ductus Arteriosus; ToF—Tetralogy of Fallot; VSD—Ventricular Septal Defect.

**Figure 3:**
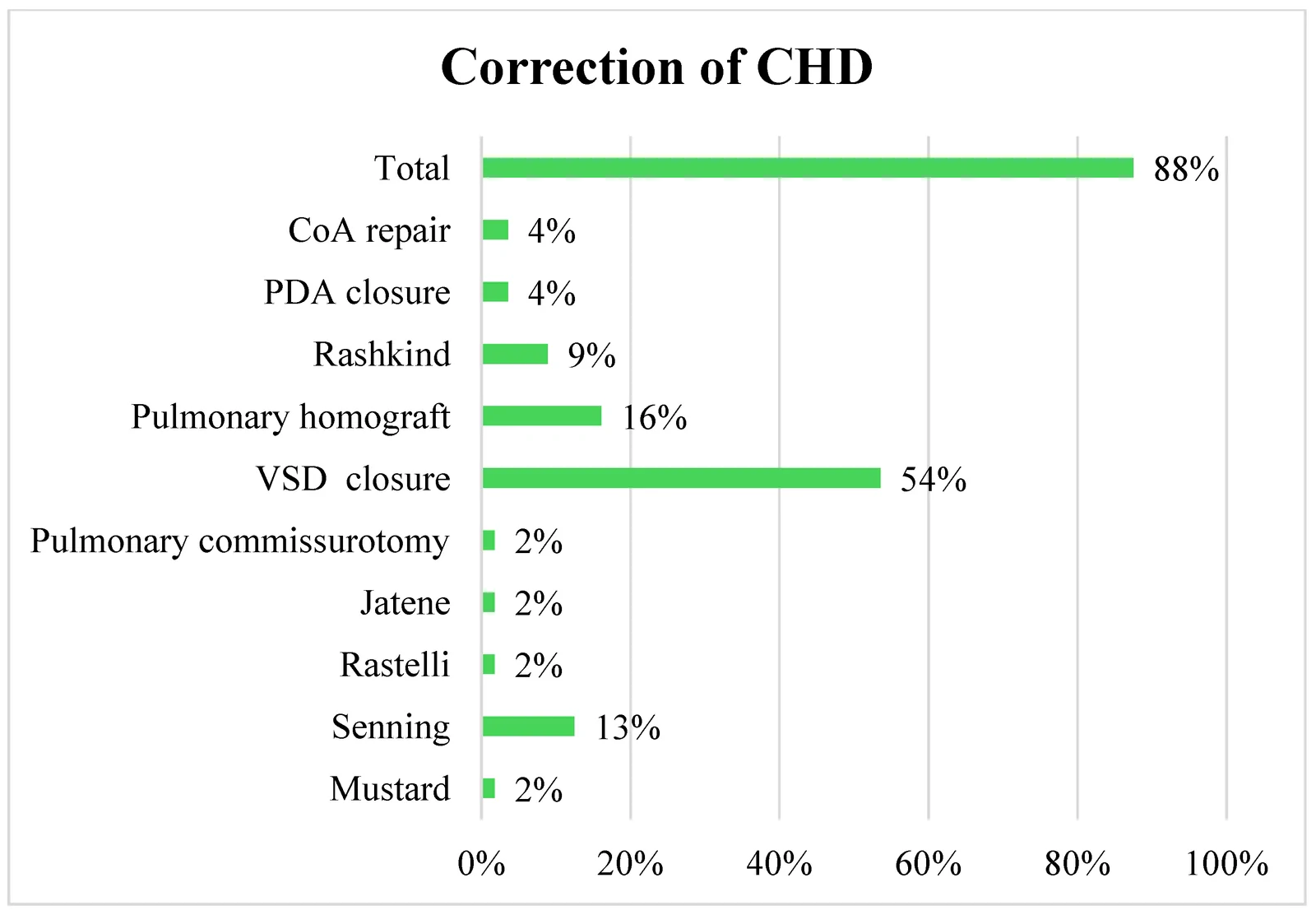
History of CHD correction. Abbreviations: CHD—Congenital Heart Disease; CoA—Coarctation of the Aorta; PDA—Patent Ductus Arteriosus; VSD—Ventricular Septal Defect.

The study cohort comprised 56 patients, including 41 males (73.2%), at a median age of 37.5 years (IQR 30.7–49.2; Figure 1). Baseline patient characteristics and follow-up data are presented in Table 1. Table 1 is presented solely to describe the two groups. A total of 154 baseline comparisons are reported; none has been adjusted for multiplicity, and all *p*-values in Table 1 should be regarded as descriptive and exploratory, with no inference intended. Applying the Benjamini–Hochberg procedure across these comparisons yielded a smallest adjusted q-value of 0.83, with no comparison approaching significance. The most common ACHDs were ventricular septal defect (37.5%), tetralogy of Fallot (33.9%), and atrial septal defect (8.9%). Complex ACHD, defined as the presence of more than one congenital heart defect, was identified in 28 patients (50.0%) (Figure 2). A total of 49 patients (87.5%) had a history of ACHD correction (Figure 3). Twenty-two patients (39.3%) underwent ICD implantation for primary prevention of SCD in accordance with the current guidelines [4] and individual clinical judgement.

### 3.2. Predictors of Appropriate ICD Intervention

Twenty-four patients experienced appropriate ICD interventions during follow-up (14 due to ventricular tachycardia, 4 due to ventricular fibrillation, 2 due to both types of ventricular tachyarrhythmia, and 4 with an unknown mechanism) over a median follow-up duration of 51.9 months (IQR 26.1–101.2). Follow-up duration was significantly longer in patients who experienced appropriate ICD intervention (*p* = 0.027). The median time to the first appropriate ICD intervention was 7.9 months (IQR 0.1–26.9). A similar proportion of patients underwent one or more device-related procedures during follow-up, including generator replacement, lead extraction or implantation, and device upgrade to cardiac resynchronisation therapy (*p* > 0.05).

After correction of the time origin, univariable Cox analysis identified a history of atrial fibrillation (HR 2.75, 95% CI 1.22–6.23; *p* = 0.015) and cardiomegaly on chest radiographydiamete (HR 3.99, 95% CI 1.28–12.40; *p* = 0.017), as the largest unadjusted associations with appropriate ICD intervention (Table 2); neither remained significant after the Benjamini–Hochberg correction (q ≈ 0.10), and both should therefore be regarded as unstable, exploratory associations rather than established predictors. In contrast, individual congenital lesions and markers of disease severity—including complex or cyanotic disease, a systemic right ventricle and systemic ventricular dysfunction—were not associated with the endpoint (all *p* > 0.3). Hypothyroidism was present in only two patients, both of whom had an event, and hyperthyroidism was present in only four patients; Firth-penalised Cox analysis confirmed that these estimates remained large with extremely wide confidence intervals (hypothyroidism HR 12.0, 95% CI 2.3–44.0; hyperthyroidism HR 6.1, 95% CI 1.6–18.3). Two pre-specified parsimonious models were constructed (Table 3). In the primary model combining atrial fibrillation and radiographic cardiomegaly, cardiomegaly remained the dominant marker (HR 3.47, 95% CI 1.08–11.15), with a bootstrap optimism-corrected Harrell’s C-index of 0.63; thyroid variables were deliberately excluded from all multivariable models. The effect of atrial fibrillation was non-proportional (Schoenfeld *p* = 0.009): it was negligible during the first year (HR 0.45) but pronounced thereafter (HR 6.32, 95% CI 1.54–25.90 beyond 12 months, adjusted for radiographic cardiomegaly), consistent with atrial fibrillation marking progressive remodelling whose arrhythmic expression accrues over time. The 12-month landmark used to describe this time-varying effect was not pre-specified; it was adopted after the proportional hazards assumption was violated and represents a conventional clinical landmark rather than a data-optimised threshold. This time split is therefore exploratory.

**Figure 4:**
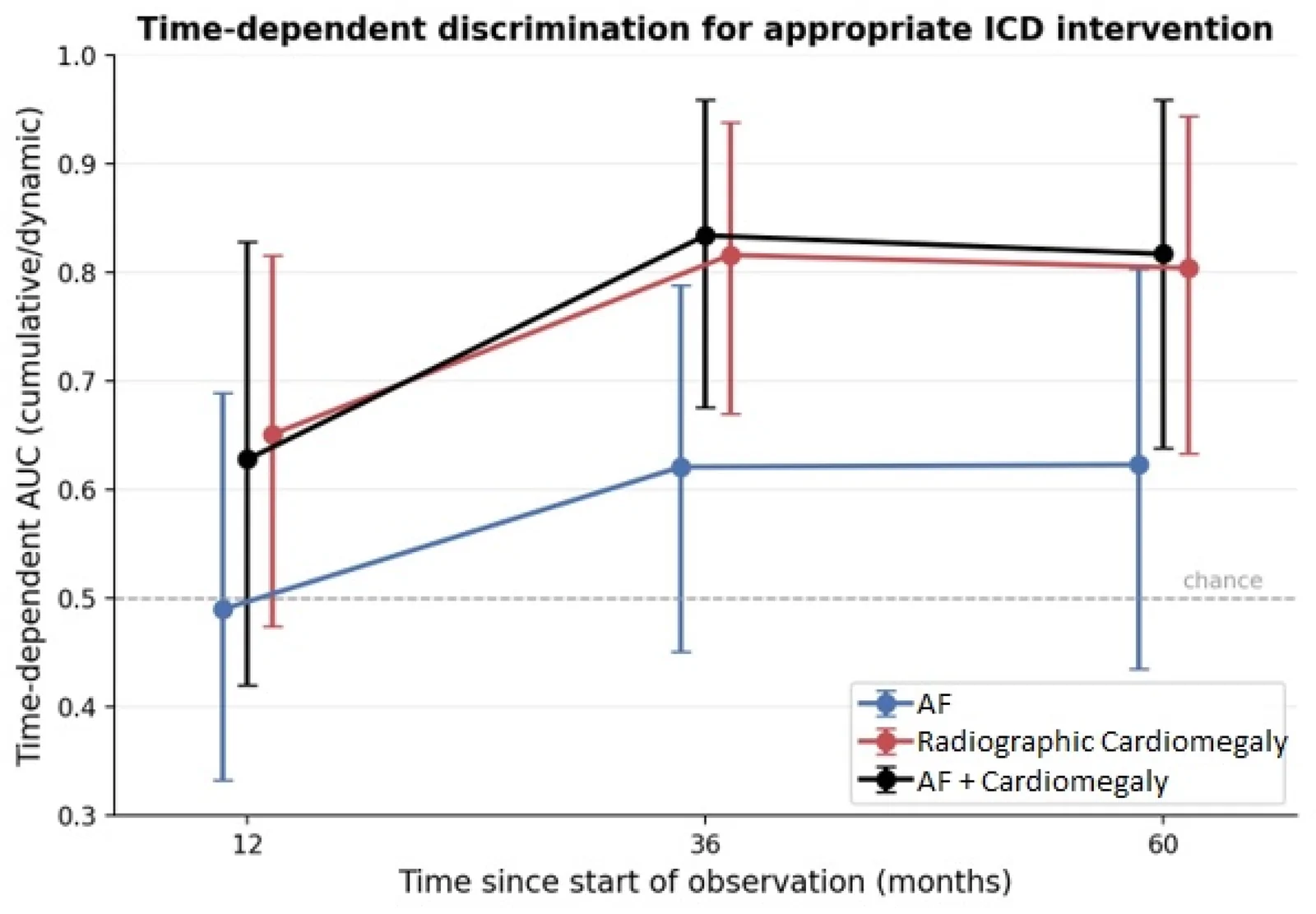
Time-dependent AUC (cumulative/dynamic) at 12, 36 and 60 months for atrial fibrillation (AF), radiographic cardiomegaly and the two-variable model (AF plus cardiomegaly) in prediction of appropriate ICD intervention. Bars denote 95% bootstrap confidence intervals. Ordinary ROC curves were not used because they are not valid for censored time-to-event outcomes. AUC—area under the curve.

**Figure 5:**
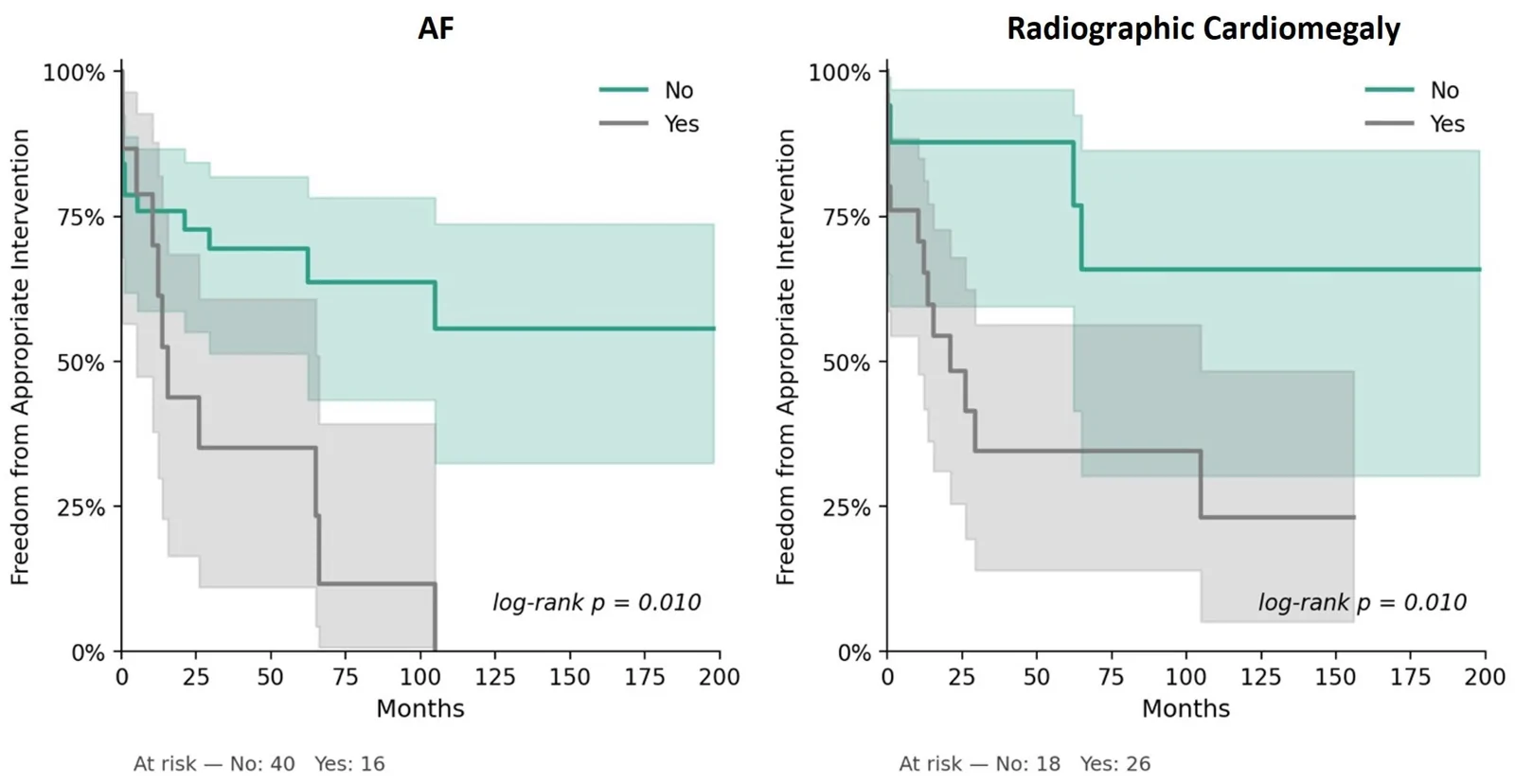
Kaplan–Meier curves for freedom from appropriate ICD intervention according to atrial fibrillation—AF (**left**) and cardiomegaly on chest radiograph (**right**). Log-rank *p* = 0.010 for both. The atrial septal defect and hypothyroidism panels were removed; hypothyroidism (*n* = 2) produced an unstable, separation-driven estimate.

Because ordinary ROC curves are not valid for censored time-to-event data, discrimination was assessed with time-dependent AUC (Figure 4). Discrimination was modest and improved with follow-up, from approximately 0.65 at 1 year to 0.80 at 3–5 years, driven mainly by radiographic cardiomegaly; the corresponding values for the two-variable model were 0.63, 0.83 and 0.82, although the confidence intervals were wide (for example, 0.68–0.96 for the two-variable model at 3 years). Kaplan–Meier analysis showed worse freedom from appropriate ICD intervention in patients with atrial fibrillation and in those with cardiomegaly (log-rank *p* = 0.010 for both; Figure 5). When death and heart transplantation were treated as competing events, the cumulative incidence of appropriate ICD intervention was approximately 39% at 5 years. The direction of both associations was preserved across sensitivity analyses, although their precision was sensitive to the handling of time-zero events and of missing cardiomegaly data. In univariable analyses excluding the patients whose intervention coincided with the start of observation, both remained significant (atrial fibrillation HR 4.01, 95% CI 1.60–10.02; cardiomegaly HR 3.46, 95% CI 1.08–11.11); however, in the adjusted two-variable model excluding these patients, and after multiple imputation of the missing cardiomegaly values, the hazard ratios attenuated to approximately 2–3 and were no longer statistically significant. The associations are therefore directionally consistent but imprecise. These metrics indicate at most a modest, group-level association and do not support use of these markers for individual risk stratification.

### 3.3. Device-Related Complications

Periprocedural complications occurred in seven patients (12.5%) and included haematoma (four patients), pneumothorax (one patient), prolonged intubation (one patient), and fever (one patient). Among these patients, five required antibiotic treatment, while one patient with a post-procedural haematoma developed a concomitant pleural effusion.

### 3.4. Inappropriate ICD Intervention

Inappropriate ICD intervention occurred in 11 patients (19.6%) and was most frequently caused by supraventricular arrhythmias. The median time to the first inappropriate ICD intervention was 43.0 months (IQR 7.1–138.0). Univariable Cox proportional hazards analysis identified three significant predictors of inappropriate ICD intervention: antiarrhythmic drug use, body mass index (BMI) and alanine aminotransferase level (Table 4).

**Figure 6:**
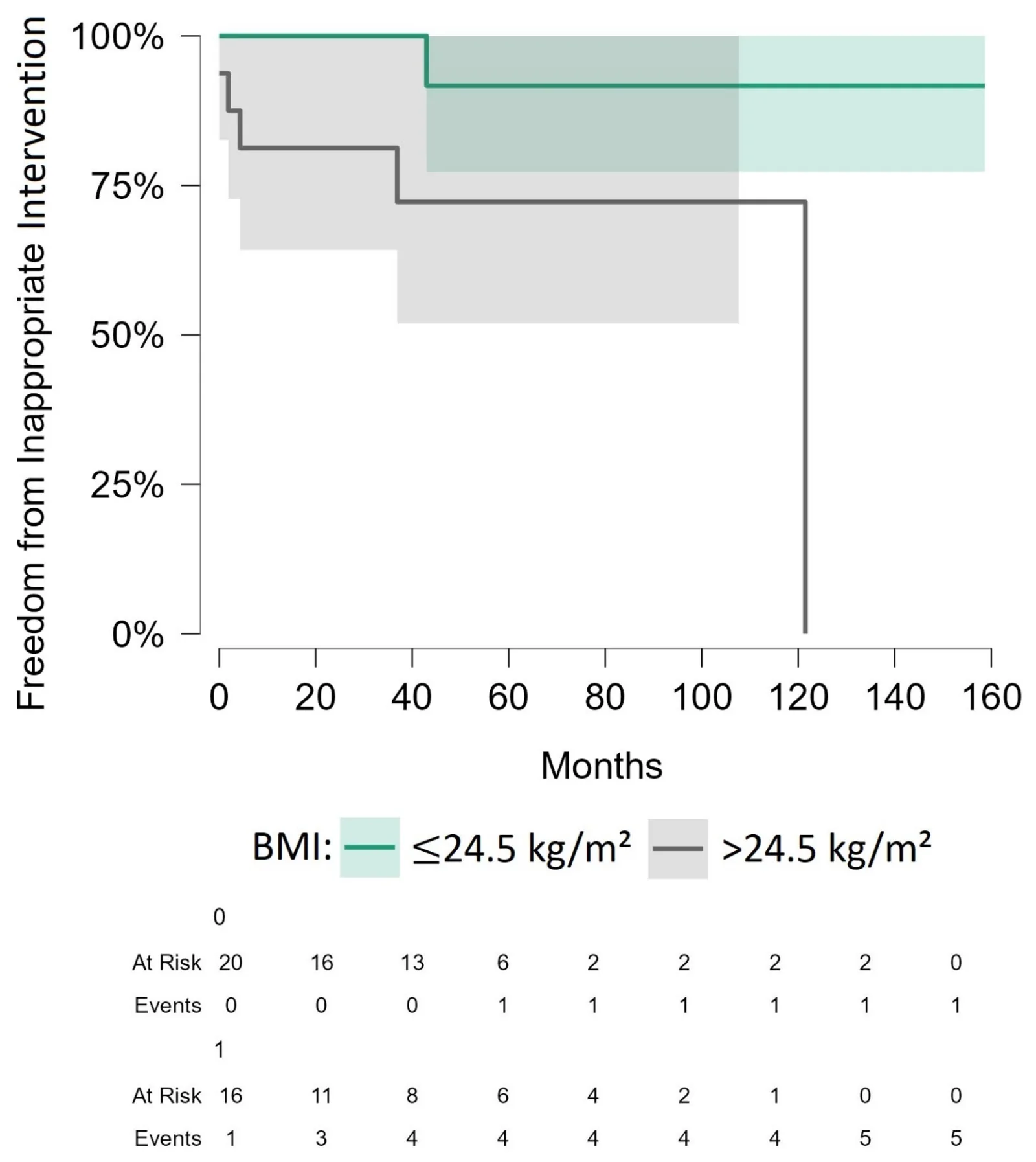
Kaplan–Meier curve for inappropriate device intervention according to the optimal body mass index (BMI) cut-off value (>24.5 kg/m^2^). *p*-value (log-rank) = 0.032. The body mass index threshold was derived from these data (a data-driven optimal cut-off) and is therefore exploratory and prone to optimism; it requires external validation.

Sick sinus syndrome (two patients, one event) produced quasi-complete separation and is not reported as an unpenalised estimate. In a penalised multivariable model, neither body mass index (HR 1.14, 95% CI 0.98–1.33) nor sick sinus syndrome (HR 6.44, 95% CI 0.60–68.83) remained significantly associated with inappropriate ICD intervention (Table 5); no independent predictor of inappropriate intervention was established. Kaplan–Meier analysis revealed that a BMI > 24.5 kg/m^2^ was associated with worse survival free from inappropriate device intervention (Figure 6). Because only 11 inappropriate interventions were observed, this model is likewise exploratory and at risk of overfitting, and the body mass index threshold in particular should be interpreted with caution given its missing data.

### 3.5. Adverse Events

Other device-related adverse events during follow-up were observed in 14 patients (25.0%) and included infective endocarditis, systemic or device pocket infections, lead damage, elevated pacing or defibrillation thresholds, diaphragmatic stimulation, and device pocket haematoma. During follow-up, five patients (8.9%) died as a result of heart failure progression, cardiac surgical procedures, or unknown causes, and one patient (1.8%) underwent heart transplantation.

## 4. Discussion

Accurate risk assessment of SCD is crucial for the appropriate management of patients with CHD, even in the absence of a prior history of ventricular arrhythmias (VAs) [5]. Our study included a wide spectrum of ACHDs with diverse indications for ICD implantation. An appropriate ICD intervention identifies a patient in whom the device detected and treated a ventricular tachyarrhythmia. It is a device-defined event rather than an adjudicated aborted sudden death and does not by itself establish net clinical benefit: appropriate therapies occur considerably more often than sudden death in the control arms of defibrillator trials, and a substantial proportion of treated episodes terminate spontaneously when detection is delayed [6,7,8,9,10,11]. Although the absence of ICD intervention does not preclude the future risk of SCD, identifying predictors of SCD during follow-up may improve clinical awareness in uncertain cases and allow for postponement of ICD implantation until clear indications emerge.

Another important consideration is the patient’s perspective on quality of life after ICD implantation. Fear of ICD intervention, particularly shocks, encourages efforts to define clear and robust indications for implantation so that a high-energy device can be recommended with the greatest possible certainty when truly indicated [12,13].

ICDs have been shown to improve prognosis in patients with heart failure and reduced ventricular systolic function, even in the absence of documented ventricular arrhythmias at baseline [14]. Vehmeijer et al. identified significant risk factors for sudden cardiac death across various types of ACHD as well as following their surgical correction [15]. A previously published meta-analysis reported that 24% of ACHD patients with an implanted ICD experienced appropriate interventions, while 25% received inappropriate shocks; other complications, particularly those related to leads, were observed in 26% of patients. Therefore, an individualised assessment of the risk–benefit profile of ICD intervention remains essential in this patient population [5].

In this cohort, most baseline parameters did not differ between patients with and without appropriate ICD intervention, and individual congenital lesions and conventional markers of disease severity were not associated with the endpoint. This pattern—an anatomically heterogeneous population in which lesion-specific descriptors carry little prognostic information—argues for lesion-agnostic markers of global cardiac remodelling, such as atrial fibrillation and radiographic cardiomegaly, rather than for lesion-specific risk models that the present sample size cannot support. Patients who received appropriate intervention had a longer follow-up, which may partly reflect greater opportunity for an event; however, the median time to first appropriate intervention was short (7.9 months), indicating that most interventions occurred well before the median follow-up of patients without intervention.

Unexpectedly, a secondary prevention indication was not significantly associated with appropriate intervention (HR 1.81, 95% CI 0.72–4.57) and remained non-significant when modelled together with atrial fibrillation (HR 1.99, 95% CI 0.78–5.08). Because a prior sustained ventricular arrhythmia is among the strongest established predictors of recurrent interventions, this null result most likely reflects limited statistical power: secondary prevention devices predominated (34 of 56 patients), reducing the contrast with the primary prevention group, and both the indication and the time origin were partly misclassified in patients implanted before referral. We therefore interpret this finding as a limitation of power rather than as evidence that the prevention indication is unimportant in ACHD.

Defibrillating lead dysfunction may influence ICD intervention outcomes [10,16]. However, follow-up device interrogation in our cohort revealed no differences in defibrillation lead coil parameters, reducing the likelihood that lead undersensing affected outcomes in patients without appropriate ICD intervention or that oversensing affected outcomes in those with inappropriate intervention.

Atrial fibrillation is a plausible marker of advanced atrial and ventricular remodelling, surgical scar burden and haemodynamic overload, and its association with appropriate ICD intervention—strongest in the later years of follow-up—is consistent with this interpretation. Radiographic cardiomegaly is a pragmatic, widely available index of global cardiac remodelling and may capture risk that individual echocardiographic measures miss, although it is not a standalone decision tool. Although hypothyroidism and hyperthyroidism can promote arrhythmia biologically, in this cohort, they were present in only two and four patients, respectively, and the corresponding hazard ratios were statistically unstable and attenuated markedly under penalised estimation; these observations are therefore hypothesis-generating only and should not influence clinical decisions. The mechanisms underlying inappropriate intervention appear distinct, with oversensing more common in patients with sick sinus syndrome and higher body mass index; because antiarrhythmic drug use is confounded by the arrhythmia burden that motivates it, it should be regarded as a marker rather than a cause.

Several limitations should be acknowledged. This was a single-centre, retrospective study without a non-ACHD control group, and the population was anatomically heterogeneous. Most importantly, only 24 appropriate interventions were available, which strictly limits the number of predictors that can be modelled and the precision of every estimate; accordingly, no association survived formal correction for multiple testing, and the analyses should be read as hypothesis-generating. Despite the observed associations, the model demonstrated only moderate predictive performance (Harrell’s C-index = 0.63); therefore, the obtained results should not be used for individual risk stratification. Several candidate predictors were present in very few patients—most notably thyroid disorders—so that the associated hazard ratios are unstable and were retained only as exploratory, penalised estimates. The start of observation preceded device implantation in some patients and coincided with the first recorded intervention in a few others; although we addressed this with a consistent time origin, delayed-entry modelling and sensitivity analyses, residual immortal time and survivorship bias cannot be excluded. Some variables, including radiographic cardiomegaly and QRS duration, had appreciable missing data. Right-ventricular systolic pressure, for example, was estimable in only 19 of 56 patients, so the apparently lower values in the intervention group in Table 1 rest on a handful of measurements and should not be interpreted as protective. The lack of significant associations for individual ACHDs does not imply that the type of defect is clinically irrelevant; rather, it most likely reflects the heterogeneity of the study population and the limited number of observed events. Finally, appropriate ICD intervention is a clinically meaningful but imperfect surrogate for sudden cardiac death, and its absence does not exclude future arrhythmic risk; these results require validation in larger, multicentre ACHD cohorts before they can inform individual decisions about ICD implantation. Two further limitations deserve emphasis. First, every patient in this cohort had already received a defibrillator, so the study population is conditioned on the implantation decision itself. The associations reported here describe arrhythmic risk after implantation and cannot be extrapolated to the question of which patients with congenital heart disease should receive an ICD; models addressing that question have been developed in unimplanted populations [17,18]. Second, we lacked data on device programming, on catheter ablation, and on changes in antiarrhythmic therapy during follow-up. Devices from four manufacturers were programmed at operator discretion, and detection settings were not standardised or systematically retrievable. Each of these factors materially influences the number of therapies delivered: in randomised programming trials, detection zones and delays alone altered appropriate therapy rates several-fold [19,20,21,22,23], antitachycardia pacing terminates the majority of fast ventricular tachycardias that would otherwise be shocked [24,25,26], and both catheter ablation and amiodarone substantially reduce subsequent device therapies [27,28,29,30,31,32]. Absolute intervention rates in this cohort should therefore not be compared directly with cohorts programmed or managed under different conventions, and the endpoint should be understood as device- and management-dependent. No randomised trial of ICD programming has been conducted in an ACHD population.

## 5. Conclusions

In a heterogeneous real-world ACHD cohort, a history of atrial fibrillation and a simple radiographic marker of cardiac remodelling—cardiomegaly—were associated with appropriate ICD intervention, whereas individual lesion type and disease-severity descriptors were not. Given the small number of events, the sparsity of several predictors, and the lack of any associations that remained significant after correction for multiple testing, these findings should be considered hypothesis-generating. They require validation in larger, multicentre ACHD cohorts before they can inform clinical practice. Because all patients had already received a device, these findings describe arrhythmic risk after implantation and cannot be extrapolated to the decision of whether to implant an ICD in an unselected ACHD population.

## Figures and Tables

**Table 1:** General characteristics and procedural and follow-up data. Comparison of two subgroups refers to appropriate ICD intervention occurrence.

Parameter	Total (N = 56) ^1^	Appropriate ICD Intervention (N = 24) ^1^	No Appropriate ICD Intervention (N = 32) ^1^	*p*-Value ^2^
**Demographic data**				
Male	41 (73.2%)	17 (70.8%)	24 (75.0%)	0.728
Age [years]	37.5 (30.7–49.2)	45.1 (34.1–51.2)	35.7 (29.9–43.8)	0.087
**Clinical data**				
Height [cm]	172.5 (168.0–182.1)	177.0 (170.0–184.2)	171.3 (165.7–179.0)	0.157
Weight [kg]	74.0 (61.0–84.3)	79.6 (68.0–88.0)	62.0 (56.1–80.0)	**0.021**
BMI [kg/m^2^]	24.1 (21.1–25.8)	25.5 (23.5–26.7)	23.0 (19.6–24.6)	**0.023**
Heart failure	31 (55.4%)	16 (66.7%)	15 (46.9%)	0.140
NYHA class:				
I	6 (10.7%)	5 (20.8%)	1 (3.1%)	0.074
II	15 (26.8%)	7 (29.2%)	8 (25.0%)	0.728
III	10 (17.9%)	4 (16.7%)	6 (18.8%)	1.000
Beta-blocker	52 (92.9%)	23 (95.8%)	29 (90.6%)	0.627
Antiarrhythmic drug	11 (19.6%)	8 (33.3%)	3 (9.4%)	**0.041**
Digoxin/methyldigoxin	3 (5.4%)	3 (12.5%)	0 (0.0%)	0.073
DOAC	9 (16.1%)	5 (20.8%)	4 (12.5%)	0.475
VKA	18 (32.1%)	9 (37.5%)	9 (28.1%)	0.457
Atrial fibrillation	16 (28.6%)	11 (45.8%)	5 (15.6%)	**0.013**
SVT	20 (35.7%)	10 (41.7%)	10 (31.3%)	0.421
Ventricular arrhythmia	40 (71.4%)	15 (62.5%)	25 (78.1%)	0.200
Prior catheter ablation of arrhythmia	11 (19.6%)	5 (20.8%)	6 (18.8%)	0.846
Ventricular	5 (8.9%)	2 (8.3%)	3 (9.4%)	1.000
Supraventricular	6 (10.7%)	3 (12.5%)	3 (9.4%)	1.000
Sick sinus syndrome	2 (3.6%)	0 (0.0%)	2 (6.3%)	0.501
Conduction disturbances	18 (32.1%)	6 (25.0%)	12 (37.5%)	0.322
ICD-VR	27 (49.1%)	12 (50.0%)	15 (48.4%)	0.906
ICD-DR	12 (21.8%)	6 (25.0%)	6 (19.4%)	0.615
S-ICD	3 (5.5%)	1 (4.2%)	2 (6.5%)	1.000
Epicardial lead	5 (9.1%)	2 (8.3%)	3 (9.7%)	1.000
CRT-D	10 (18.2%)	3 (12.5%)	7 (22.6%)	0.486
Primary prevention of SCD	22 (39.3%)	6 (25.0%)	16 (50.0%)	0.058
Prior pacemaker	6 (10.7%)	2 (8.3%)	4 (12.5%)	0.691
Prosthetic heart valve	11 (19.6%)	4 (16.7%)	7 (21.9%)	0.741
Coronary artery disease	6 (10.7%)	4 (16.7%)	2 (6.3%)	0.385
Myocardial infarction	3 (5.4%)	2 (8.3%)	1 (3.1%)	0.571
Hypertension	14 (25.0%)	4 (16.7%)	10 (31.3%)	0.350
Diabetes mellitus type 2	2 (3.6%)	1 (4.2%)	1 (3.1%)	1.000
Hypothyroidism	2 (3.6%)	2 (8.3%)	0 (0.0%)	0.179
Hyperthyroidism	4 (7.1%)	3 (12.5%)	1 (3.1%)	0.303
Prior infective endocarditis	7 (12.5%)	3 (12.5%)	4 (12.5%)	1.000
Systolic blood pressure [mmHg]	117.0 (108.5–127.5)	118.5 (110.0–127.3)	117.0 (105.5–125.0)	0.550
Diastolic blood pressure [mmHg]	74.0 (67.0–79.5)	75.0 (68.5–80.0)	72.0 (66.5–76.0)	0.348
Heart rate [bpm]	64.0 (58.5–77.5)	71.0 (59.3–83.0)	61.0 (57.0–70.0)	0.304
Oxygen saturation [%]	97.0 (95.0–99.0)	97.5 (96.0–99.0)	97.0 (95.0–100.0)	0.729
**Congenital heart disease**				
Ventricular septal defect	21 (37.5%)	9 (37.5%)	12 (37.5%)	1.000
Atrial septal defect	5 (8.9%)	4 (16.7%)	1 (3.1%)	0.153
Patent ductus arteriosus	2 (3.6%)	0 (0.0%)	2 (6.3%)	0.501
Tetralogy of Fallot	19 (33.9%)	8 (33.3%)	11 (34.4%)	0.935
Ebstein anomaly	3 (5.4%)	3 (12.5%)	0 (0.0%)	0.073
Coarctation of the aorta	3 (5.4%)	0 (0.0%)	3 (9.4%)	0.252
Bicuspid aortic valve	4 (7.1%)	1 (4.2%)	3 (9.4%)	0.627
d-TGA	3 (5.4%)	1 (4.2%)	2 (6.3%)	1.000
cc-TGA	3 (5.4%)	2 (8.3%)	1 (3.1%)	0.571
Taussig–Bing syndrome	3 (5.4%)	1 (4.2%)	2 (6.3%)	1.000
Atrioventricular septal defect	1 (1.8%)	1 (4.2%)	0 (0.0%)	0.429
Pulmonary atresia	2 (3.6%)	0 (0.0%)	2 (6.3%)	0.501
Subvalvular pulmonary stenosis	1 (1.8%)	0 (0.0%)	1 (3.1%)	1.000
Others	15 (26.8%)	6 (25.0%)	9 (28.1%)	0.794
Cyanotic CHD	27 (48.2%)	11 (45.8%)	16 (50.0%)	0.757
>1 CHD	28 (50.0%)	12 (50.0%)	16 (50.0%)	1.000
**Prior CHD correction**				
Mustard	1 (1.8%)	0 (0.0%)	1 (3.1%)	1.000
Senning	7 (12.5%)	1 (4.2%)	6 (18.8%)	0.219
Rastelli	1 (1.8%)	1 (4.2%)	0 (0.0%)	0.429
Jatene	1 (1.8%)	0 (0.0%)	1 (3.1%)	1.000
Pulmonary commissurotomy	1 (1.8%)	0 (0.0%)	1 (3.1%)	1.000
VSD closure	30 (53.6%)	13 (54.2%)	17 (53.1%)	0.938
Pulmonary homograft	9 (16.1%)	4 (16.7%)	5 (15.6%)	1.000
Rashkind	5 (8.9%)	1 (4.2%)	4 (12.5%)	0.379
PDA closure	2 (3.6%)	0 (0.0%)	2 (6.3%)	0.501
CoA repair	2 (3.6%)	0 (0.0%)	2 (6.3%)	0.501
Total	49 (87.5%)	20 (83.3%)	29 (90.6%)	0.447
**Electrocardiography**				
Intrinsic QRS	39 (79.6%)	17 (81.0%)	22 (78.6%)	1.000
QRS [ms]	155.0 (123.0–176.0)	155.0 (129.0–178.0)	158.0 (119.5–174.5)	0.435
QTc—Bazett [ms]	470.0 (449.0–497.0)	472.0 (456.5–500.5)	458.5 (444.8–496.5)	0.246
**Echocardiography**				
LV systolic dysfunction	30 (54.5%)	13 (54.2%)	17 (54.8%)	0.960
RV systolic dysfunction	27 (48.2%)	9 (37.5%)	18 (56.3%)	0.165
Systemic RV	15 (26.8%)	3 (12.5%)	12 (37.5%)	0.065
IVS [mm]	11.0 (9.0–12.1)	11.0 (9.0–12.2)	11.0 (10.0–12.0)	0.642
LV posterior wall [mm]	10.0 (8.5–11.0)	11.0 (9.0–11.0)	10.0 (8.0–11.0)	0.133
LVESD [mm]	40.0 (34.0–46.0)	40.0 (34.5–45.0)	40.0 (33.0–49.5)	1.000
LVEDD [mm]	51.0 (47.0–61.0)	51.0 (48.0–61.0)	54.0 (46.5–61.3)	0.811
LVEDV [mL]	165.0 (116.0–197.0)	184.0 (177.0–219.0)	145.5 (106.3–177.0)	0.136
LVEF [%]	45.0 (31.0–55.0)	47.5 (30.0–60.0)	45.0 (32.3–55.0)	0.777
RVdA [cm^2^]	46.3 (38.7–54.8)	49.5 (44.3–54.3)	44.4 (38.7–53.9)	0.524
S’ RV [cm/s]	8.0 (6.0–9.5)	9.0 (5.5–10.0)	7.0 (6.0–8.8)	0.204
TAPSE [mm]	14.5 (12.0–18.0)	16.0 (12.0–18.5)	13.0 (12.0–16.0)	0.392
GLS [%]	−8.8 (−10.8 to −7.0)	−8.3 (−9.6 to −6.2)	−9.3 (−11.3 to −7.1)	0.659
FAC [%]	21.0 (19.0–26.0)	24.0 (17.0–31.0)	21.0 (19.5–25.0)	0.889
LA [mm]	46.0 (40.0–49.0)	47.5 (43.5–49.5)	42.0 (36.0–47.0)	0.166
LAA [cm^2^]	25.5 (22.0–28.4)	26.0 (22.0–28.0)	25.0 (22.7–30.0)	0.550
RAA [cm^2^]	28.0 (21.5–32.0)	27.5 (22.8–32.0)	28.0 (20.0–32.0)	0.849
Mitral regurgitation:	34 (60.7%)	16 (66.7%)	18 (56.3%)	0.430
Moderate	2 (3.6%)	0 (0.0%)	2 (6.3%)	0.501
Severe	3 (5.4%)	1 (4.2%)	2 (6.3%)	1.000
Aortic regurgitation:	28 (50.0%)	12 (50.0%)	16 (50.0%)	1.000
Moderate	2 (3.6%)	0 (0.0%)	2 (6.3%)	0.501
Severe	1 (1.8%)	0 (0.0%)	1 (3.1%)	1.000
Tricuspid regurgitation:	43 (76.8%)	20 (83.3%)	23 (71.9%)	0.358
Moderate	9 (16.1%)	2 (8.3%)	7 (21.9%)	0.274
Moderate-to-severe	6 (10.7%)	3 (12.5%)	3 (9.4%)	1.000
Severe	4 (7.1%)	1 (4.2%)	3 (9.4%)	0.627
Pulmonary regurgitation:	27 (48.2%)	12 (50.0%)	15 (46.9%)	0.817
Moderate	2 (3.6%)	1 (4.2%)	1 (3.1%)	1.000
Moderate-to-severe	1 (1.8%)	0 (0.0%)	1 (3.1%)	1.000
Severe	5 (8.9%)	2 (8.3%)	3 (9.4%)	1.000
Ascending aorta [mm]	33.5 (30.0–40.0)	35.0 (30.0–40.0)	33.0 (27.8–39.5)	0.538
Aortic arch [mm]	30.0 (26.8–32.3)	29.0 (26.0–32.0)	31.0 (28.0–33.0)	0.594
RVSP [mmHg]	38.0 (33.5–47.5)	37.0 (31.0–40.0)	45.5 (41.8–62.3)	**0.047**
Pulmonary trunk [mm]	25.0 (22.0–30.0)	25.5 (23.8–30.8)	25.0 (22.0–30.0)	0.809
IVC [mm]	19.5 (15.8–23.0)	20.0 (15.0–23.0)	19.0 (17.0–22.5)	0.817
IVC decreased collapsibility	9 (16.7%)	4 (17.4%)	5 (16.1%)	1.000
Pericardial effusion	3 (5.6%)	0 (0.0%)	3 (9.7%)	0.253
**Holter ECG monitoring**				
VES/24 h	529.5 (85.8–1360.3)	326.0 (51.5–1898.0)	825.0 (129.0–1268.5)	1.000
nsVT/24 h	0.0 (0.0–1.0)	0.0 (0.0–1.0)	0.0 (0.0–0.5)	0.727
AF/AFL/Sustained SVT	5 (17.9%)	2 (16.7%)	3 (18.8%)	1.000
**Laboratory blood tests**				
NT-proBNP [pg/mL]	765.7 (314.1–1355.5)	837.5 (358.4–1288.0)	589.3 (299.3–1919.0)	0.675
ALT [U/L]	28.0 (18.3–44.0)	28.5 (20.3–44.8)	26.0 (17.8–41.7)	0.309
AST [U/L]	26.0 (21.8–40.8)	26.0 (22.5–43.3)	29.5 (21.3–40.0)	0.611
APTT [s]	33.2 (28.9–39.8)	34.4 (28.7–40.1)	33.1 (29.8–37.5)	0.763
Total bilirubin [mg/dL]	1.0 (0.7–1.9)	1.1 (0.7–1.3)	0.9 (0.7–2.0)	1.000
CRP [mg/dL]	0.2 (0.1–0.4)	0.2 (0.1–0.4)	0.1 (0.1–0.5)	0.659
Glucose [mg/dL]	93.1 (83.4–101.3)	94.0 (88.9–104.0)	91.3 (82.2–97.8)	0.144
Sodium [mmol/L]	141.0 (139.8–142.0)	141.0 (140.0–142.0)	141.0 (139.2–142.2)	0.526
Potassium [mmol/L]	4.4 (4.2–4.7)	4.4 (4.2–4.5)	4.5 (4.2–4.8)	0.337
Creatinine [mg/dL]	0.9 (0.8–1.0)	0.9 (0.8–1.1)	0.9 (0.8–1.0)	0.875
Total cholesterol [mg/dL]	159.9 (139.2–181.6)	159.7 (140.0–177.0)	160.1 (136.9–182.0)	0.582
Triglycerides [mg/dL]	86.0 (68.0–113.3)	85.0 (68.0–121.0)	86.4 (66.3–108.0)	0.711
HDL-C [mg/dL]	51.4 (43.0–56.8)	47.2 (43.0–60.0)	52.2 (47.3–56.5)	0.491
LDL-C [mg/dL]	104.2 (86.8–116.0)	103.7 (93.5–114.0)	105.9 (82.3–116.5)	0.699
Leukocytes [×10^3^/µL]	6.0 (5.1–6.9)	6.4 (5.1–7.1)	6.0 (5.1–6.8)	0.529
Erythrocytes [×10^6^/µL]	4.8 (4.5–5.1)	4.9 (4.5–5.1)	4.8 (4.4–5.0)	0.461
Haemoglobin [g/dL]	14.5 (13.6–15.5)	15.1 (14.2–15.7)	14.2 (13.4–15.1)	0.119
Haematocrit [%]	42.9 (40.0–45.6)	44.2 (40.7–46.2)	41.7 (39.9–44.9)	0.132
MCV [fL]	89.8 (86.4–92.7)	90.7 (86.9–94.3)	89.0 (86.3–92.0)	0.253
Thrombocytes [×10^3^/µL]	180.0 (158.3–211.5)	179.0 (158.3–208.0)	183.5 (157.5–227.5)	0.797
RDW-SD [fL]	41.2 (39.9–45.4)	41.5 (39.7–45.8)	41.1 (40.0–43.6)	0.721
Neutrophils [×10^3^/µL]	3.5 (2.8–4.6)	3.1 (2.6–4.6)	3.5 (3.0–4.4)	0.625
Lymphocytes [×10^3^/µL]	1.7 (1.3–2.0)	1.8 (1.6–2.4)	1.5 (1.2–1.8)	**0.019**
INR	1.2 (1.1–1.4)	1.2 (1.1–1.5)	1.1 (1.1–1.4)	0.838
TSH [µIU/mL]	1.8 (1.2–3.4)	1.5 (1.0–2.4)	2.0 (1.4–4.0)	0.105
Troponin T [ng/L]	10.3 (6.4–16.0)	8.6 (6.4–12.2)	10.4 (6.7–18.1)	0.839
**Chest radiograph**				
Pulmonary circulatory congestion	12 (27.3%)	6 (33.3%)	6 (23.1%)	0.453
Pleural effusion	4 (9.1%)	0 (0.0%)	4 (15.4%)	0.133
Cardiomegaly	26 (59.1%)	14 (77.8%)	12 (46.2%)	0.061
**Follow-up—clinical**				
Periprocedural complications	7 (12.5%)	3 (12.5%)	4 (12.5%)	1.000
Follow-up time [months]	51.9 (26.1–101.2)	71.1 (46.2–107.8)	41.0 (8.5–76.2)	**0.027**
1 CIED procedure during follow-up	16 (28.6%)	7 (29.2%)	9 (28.1%)	0.932
>1 CIED procedure during follow-up	8 (14.3%)	5 (20.8%)	3 (9.4%)	0.268
Death	5 (8.9%)	3 (12.5%)	2 (6.3%)	0.642
Heart transplantation	1 (1.8%)	0 (0.0%)	1 (3.1%)	1.000
**Follow-up—device**				
Coil sensing [mV]	10.1 (6.5–12.0)	8.7 (5.6–12.0)	10.6 (7.7–11.8)	0.664
Coil impedance [Ω]	450.0 (399.3–555.3)	427.0 (399.0–522.3)	478.0 (413.0–620.0)	0.368
High voltage [Ω]	66.0 (54.5–75.0)	75.0 (53.8–79.3)	64.0 (57.0–71.0)	0.251
Inappropriate ICD interventions:	11 (19.6%)	6 (25.0%)	5 (15.6%)	0.382
Supraventricular arrhythmia	6 (54.5%)	3 (50.0%)	3 (60.0%)	1.000
Unknown	5 (45.5%)	3 (50.0%)	2 (40.0%)	1.000
Other adverse event (device-related)	14 (25.0%)	5 (20.8%)	9 (28.1%)	0.533

^1^ Median (Q1-Q3); N (%); ^2^ Wilcoxon’s rank sum test; Pearson’s chi-squared test (Fisher’s exact test for N < 5). Abbreviations: AF—Atrial Fibrillation; AFL—Atrial Flutter; ALT—Alanine Aminotransferase; APTT—Activated Partial Thromboplastin Time; AST—Aspartate Aminotransferase; BMI—Body Mass Index; cc-TGA—Congenitally Corrected Transposition of the Great Arteries; CHD—Congenital Heart Disease; CIED—Cardiac Implantable Electronic Device; CoA—Coarctation of the Aorta; CRP—C-reactive Protein; CRT-D—Cardiac Resynchronisation Therapy with Defibrillator; DOAC—Direct Oral Anticoagulant; d-TGA—Dextro-Transposition of the Great Arteries; FAC—Fractional Area Change; GLS—Global Longitudinal Strain; HDL-C—High-Density Lipoprotein Cholesterol; ICD—Implantable Cardioverter-Defibrilator; ICD-DR—Dual-Chamber ICD; ICD-VR—Single-Chamber ICD; INR—International Normalised Ratio; IVC—Inferior Vena Cava; IVS—Interventricular Septum; LA—Left Atrium; LAA—Left-Atrial Area; LDL-C—Low-Density Lipoprotein Cholesterol; LV—Left Ventricle; LVEDD—Left-Ventricular End-Diastolic Diameter; LVEDV—Left-Ventricular End-Diastolic Volume; LVEF—Left-Ventricular Ejection Fraction; LVESD—Left-Ventricular End-Systolic Diameter; MCV—Mean Cell Volume; nsVT—Non-sustained Ventricular Tachycardia; NT-proBNP—N-terminal pro-B-type Natriuretic Peptide; NYHA—New York Heart Association; PDA—Patent Ductus Arteriosus; QTc—Corrected QT Interval; RAA—Right Atrial Area; RDW-SD—Red Blood Cell Distribution Width—Standard Deviation; RV—Right Ventricle; RVdA—Right-Ventricular Diastolic Area; RVSP—Right-Ventricular Systolic Pressure; SCD—Sudden Cardiac Death; S-ICD—Subcutaneous ICD; SVT—Supraventricular Tachycardia; TAPSE—Tricuspid Annular Plane Systolic Excursion; TSH—Thyroid-Stimulating Hormone; VES—Ventricular Extrasystole; VKA—Vitamin K Antagonist; VSD—Ventricular Septal Defect. Between-group *p*-values are descriptive and were not corrected for multiple comparisons; they should not be interpreted as identifying independent predictors. Statistically significant values <0.05 have been bolded.

**Table 2:** Univariable Cox proportional hazards models for appropriate device intervention. Estimates were re-derived on a single, consistently defined time origin, and the full pre-specified predictor set is shown. Thyroid disorders are sparse (hypothyroidism *n* = 2, both with events, giving quasi-complete separation; hyperthyroidism *n* = 4) and their estimates are exploratory; body mass index (20/56 missing) and radiographic cardiomegaly (12/56 missing) had incomplete data. No predictor remained significant after the Benjamini–Hochberg correction. Hazard ratios shown for the thyroid disorders are unpenalised maximum-likelihood estimates.

Parameter	Non-Missing N	Exposed N	Events Among Exposed	Hazard Ratio	95% Confidence Interval	*p*-Value
Body mass index [kg/m^2^]	36	-	-	1.10	1.01–1.19	**0.039**
Antiarrhythmic drug	56	11	8	2.32	0.96–5.21	0.062
Atrial fibrillation	56	16	11	2.79	1.23–6.22	**0.015**
Radiographic cardiomegaly	44	26	14	3.66	1.33–12.25	**0.011**
Hypothyroidism	56	2	2	12.04	2.26–44.03	**0.007**
Hyperthyroidism	56	4	3	6.09	1.55–18.28	**0.013**
Secondary prevention of sudden cardiac death	56	34	18	1.69	0.72–4.50	0.234

Statistically significant values <0.05 have been bolded.

**Table 3:** Multivariable Cox proportional hazards models for appropriate device intervention. Models were revised to exclude hypothyroidism (*n* = 2), which is statistically unstable. Model 1 (atrial fibrillation + cardiomegaly): Harrell’s C 0.645, optimism-corrected 0.627. Model 2 (atrial fibrillation + secondary prevention): Harrell’s C 0.614. Maximum two covariates per model given 24 events.

Parameters	Hazard Ratio	95% Confidence Interval	*p*-Value
Atrial fibrillation Radiographic cardiomegaly	1.77	0.67–4.66	0.251
	3.47	1.08–11.15	**0.037**
Atrial fibrillation Secondary prevention of SCD	2.91	1.29–6.59	**0.010**
	1.99	0.78–5.08	0.148

Statistically significant values <0.05 have been bolded.

**Table 4:** Univariable Cox proportional hazards models for inappropriate device intervention. Estimates use the corrected time origin.

Parameter	Non-Missing N	Exposed N	Events Among Exposed	Hazard Ratio	95% Confidence Interval	*p*-Value
Body mass index [kg/m^2^]	36	-	-	1.22	1.01–1.48	**0.039**
Antiarrhythmic drug	56	11	8	4.57	1.13–18.46	**0.033**
Alanine aminotransferase [U/L]	50	-	-	1.04	1.00–1.08	**0.036**

Statistically significant values <0.05 have been bolded.

**Table 5:** Multivariable Cox proportional hazards model for inappropriate device intervention. With only 11 inappropriate interventions and sick sinus syndrome affecting 2 patients, the unpenalised model is non-identifiable; penalised estimates are shown, and neither covariate remained significant. This model is exploratory.

Parameters	Hazard Ratio	95% Confidence Interval	*p*-Value
Body mass index [kg/m^2^]	1.14	0.98–1.33	0.095
Sick sinus syndrome	6.44	0.60–68.83	0.123

## Data Availability

The data are available on request from the corresponding author (JM).
